# The Role of Health Concerns in Phishing Susceptibility: Survey Design Study

**DOI:** 10.2196/18394

**Published:** 2020-05-04

**Authors:** Mohamed Abdelhamid

**Affiliations:** 1 Department of Information Systems College of Business California State University Long Beach Long Beach, CA United States

**Keywords:** phishing, health concerns, disposition to trust, risk-taking propensity, cybercrime, security, internet, trust, risk-taking, crime victims

## Abstract

**Background:**

Phishing is a cybercrime in which the attackers usually impersonate a trusted source. The attackers usually send an email that contains a link that allows them to steal the receiver’s personal information. In the United States, phishing is the number one cybercrime by victim count according to the Federal Bureau of Investigation’s 2019 internet crime report. Several studies investigated ways to increase awareness and improve employees’ resistance to phishing attacks. However, in 2019, successful phishing attacks continued to rise at a high rate

**Objective:**

The objective of this study was to investigate the influence of personality-based antecedents on phishing susceptibility in a health care context.

**Methods:**

Survey data were collected from participants through Amazon Mechanical Turk to test a proposed conceptual model using structural equation modeling.

**Results:**

A total of 200 participants took part. Health concerns, disposition to trust, and risk-taking propensity yielded higher phishing susceptibility. This highlights the important of personality-based factors in phishing attacks. In addition, females had a higher phishing susceptibility than male participants

**Conclusions:**

While previous studies used health concerns as a motivator for contexts such as sharing personal health records with providers, this study shed light on the danger of higher health concerns in enabling the number one cybercrime.

## Introduction

### Background

Phishing refers to an internet cybercrime where a normal computer or mobile phone user is targeted by a cybercriminal through email. This communication is mostly intended to lure the user to provide their sensitive data such as personal health care records, passwords, bank information, and passwords. In a phishing attack, the attacker sends an email that impersonates a legitimate organization or person. The cybercriminal uses social engineering techniques to encourage the receiver to click on a suspicious link. The link can download a malicious app or provide a form that asks the recipient to enter sensitive personal information. Phishing attacks can target individuals, employees, corporations, or governments. Attackers are motivated by many factors such as achieving financial benefits or gaining a reputation in the cybercriminal community. An attacker can sell a stolen personal health care record for thousands of dollars [1].

According to the US Federal Bureau of Investigation’s annual Internet Crime Complaint Center report for 2019 [2], there were about US $3.5 billion in financial losses alone due to cases of theft, fraud, and exploitation on the internet. The report states that the most prevalent type of attack was the category Phishing/Vishing/Smishing/Pharming, various terms that are used to define different means to phish. For example, fraud conducted over the phone is termed *vishing*. *Smishing* refers to attacks conducted using texting. Phishing was ranked first by the number of victims, with more than twice as many victims as the second-ranked type of attacks. Phishing attacks have been increasing significantly in the past few years [3]*.*

A recent IBM Security report ranked the health care industry first in term of the average cost of a data breach [4]. The report stated that phishing was one of the most common methods used in carrying out an attack. In 2018, about 15 million patients’ records were breached in the United States. In addition, in the first 6 months of 2019, 25 million patients were compromised with phishing being the main factor behind most breaches [5].

Phishing not only has a negative influence on individuals’ monetary assets but also builds doubt every single time they are contacted via phone calls, texts, or emails. For an individual, this shatters the reliability of electronic media to carry out a variety of tasks. This poses a great danger to digitizing traditional paper-based patient records.

According to various recent reports and research, the most common features of phishing emails are that they are astonishingly true, and have a sense of urgency (pushing the recipient to take an action as soon as possible), too many links, unexpected attachments, and an anonymous or unknown sender. Researchers and experts have been developing and testing ways of raising users’ awareness so that they can detect phishing attacks.

However, efforts in raising awareness are yet to prove successful. As mentioned above, the success rate of the attacks has increased globally. About 35% of individuals do not even know what phishing is [6]. Many recent studies and current training focus on recommendations that many attackers get around. For example, Jensen et al [7] focused on training and recommendations that help individuals to avoid phishing. One of the main recommendations was to look for “https” in the address bar. Many online materials and articles list similar recommendations, including an unknown sender, generic greetings, and grammatical mistakes. However, according to a 2019 phishing trends and intelligence report, about 50% of phishing attacks use secure socket layer, making them harder to detect [8].

More importantly, attackers have constantly advanced and changed strategies. In fact, 2019 was a year of phishing evolution. Microsoft released a report in December 2019 that talked about evolving methods of phishing and explained the 3 most notable attack techniques of phishing they observed with their Microsoft Threat Protection services in 2019 [9].

Phishing is ultimately a social tactic. According the Verizon data breach investigation report [10], 43% of cyber attacks encompass social tactics, and of those that use social tactics, 93% are phishing attacks. Therefore, constant efforts should be invested in understanding the social and personal characteristics of individuals and victims. Detection, awareness, and training strategies need to be constantly evolving. In addition, one-size-fits-all recommendations and strategies will not benefit most individuals, organizations, or industries.

### Literature Review

Research related to phishing can be categorized into two main streams. The first stream of research investigates technical aspects that can automatically detect phishing attacks using various methods such as machine learning and text mining. Jain and Richariya [11] proposed that a Web browser can also be trained to screen emails.

Methods such as text clustering, text mining, topic modelling, and classification have also been used to improve systems that detect and block phishing emails. For example, Basavaraju and Prabhakar [12] used cluster analysis to detect spam emails. Jeeva and Rajsingh [13] applied association rule mining techniques to detect phishing emails that contain malicious links.

Niakanlahiji et al [14] proposed a framework of machine learning to detect phishing webpages. The framework used 15 novel features that can be used on a webpage effectively without relying on search engines or other services. Some researchers have focused on improving text mining and data extraction techniques, which are then used as scripts to extract data from emails in a semiautomatic manner and analyze them to find patterns and other data [15].

However, in 2019, about 30% of phishing emails still bypassed security detection measures [16]. Phishing volume increased by approximately 41% in 2018, and the success rate of attacks has also increased. Although phishing detection technology is advancing, it cannot keep up with the advancement rate of phishing attacks. Therefore, many experts and researchers emphasize the importance of improving user awareness and training in regard to phishing. Most experts agree that the best way to defend against phishing attacks is to train employees and individuals to detect phishing emails in addition to security measures that detect some of the attacks automatically.

Therefore, the second stream of research has focused on the user side of the equation. User awareness of phishing emails stands to be one of the main prevention measures for phishing all around cyberspace. Hence, various awareness campaigns such as public announcements, seminars, and podcasts help to make users aware of these attacks and hence prevent phishing attacks in any form [17]. Miranda [18] suggested that phishing training programs can increase employee resistance to phishing attacks. Various studies proposed a game design framework to prevent many phishing attacks [3,19]. This framework enhances user avoidance behavior through motivation and hence results in preventing these phishing attacks. Other researchers looked at the characteristics of victims such as their email habits, perception of risk, and self-efficacy [20,21].

However, researchers have defined phishing as social engineering where an attacker attempts to fraudulently acquire sensitive information from a victim by impersonating a trustworthy third party. This brings in the scope for understanding normal users and their mentality gaps. Individuals are not always driven by rational thought and knowledge when making a decision or taking an action [22]. People may make decisions quickly and may be driven by emotions or other factors [23]. For example, Jalali et al [24] found that perceived risk is not associated with click behavior, whereas workload is positively associated with click behavior. The limited number of studies looking at the influence of individuals’ characteristics and traits on phishing susceptibility in a health care context creates a gap in the research.

### Objective

This study, motivated by improving phishing training and prevention measures by understanding individual characteristics, investigated the influence of personality-based factors—health concerns, disposition to trust, and risk taking—on phishing sustainability in health care–related attacks. The antecedents cover three areas of personality related to the context: concerns, risk, and trust. This allowed for identifying risk groups and understanding normal individuals and the mentality loopholes that an attacker uses to execute their tasks. This is one of the few studies that focused on personality-based antecedents as they relate to phishing susceptibility, specifically in the context of health care.

## Methods

### Conceptual Model

**Figure 1:**
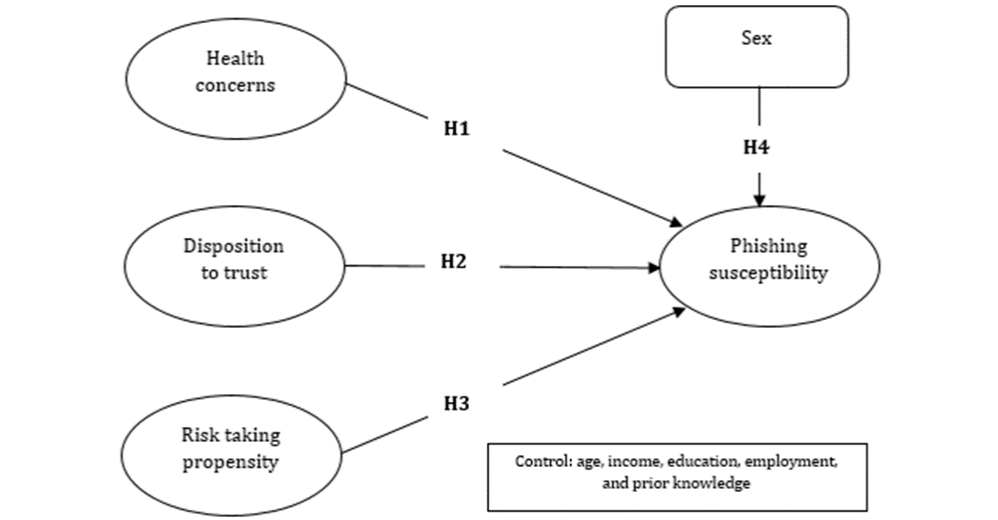
Conceptual model.

The following subsections argue for the relationships in the conceptual model shown in Figure 1. This is one the first studies that focused on personality-based variables and their influence on phishing susceptibility in the health care context.

#### Health Concerns

Health concerns refers to the degree to which an individual is concerned about their health [25]. Health-related traits have been shown to influence individuals’ choices related to behaviors affecting their health directly or indirectly [26]. People who are concerned about their health are anticipated to pursue resources as they participate in behaviors to protect their health [25]. In the context of privacy, health concerns act as a promotor to seek better health outcomes [27]. In addition, the concept of health concerns is linked with a higher likelihood to seek health information online [28]. In general, Brelsford et al [29] found that patients with a high level of health concerns are motivated to take actions related to their health. However, the construct of health concerns has not been investigated in the context of phishing.

The first study hypothesis (H1, Figure 1) was that health concerns is positively associated with phishing susceptibility.

#### Disposition to Trust

Disposition to trust is a personality construct that refers to individuals’ propensity to trust or distrust others [30]. In general, various types of trust have been extensively studied in different disciplines. The perception of trust can be linked with people or systems [31]. For example, the direct relationship between trust beliefs and use of technology has been well established [32-34]. The association between trust and information sharing has also been empirically demonstrated in previous studies [35-37]. These findings suggest that releasing personal information in exchange for e-services requires a great deal of trust. In the context of e-commerce and social media, prior literature has confirmed a positive association between trust in the system and a willingness to use the system [38,39].

The role of disposition to trust as a personality construct has been investigated in internet and website usage in general [30,40]. Bélanger and Carter [32] found that trust has an important role in promoting e-government use. Wang et al [41] investigated the effect of disposition to trust on mobile banking adoption. In addition, disposition to trust has been linked to a higher likelihood of deception [42].

The second study hypothesis (H2, Figure 1) was that disposition to trust is positively associated with phishing susceptibility.

#### Risk-Taking Propensity

A key personality aspect that influences decision making is the individual’s propensity to take on risks. An individual’s risk-taking personality is defined as the behavioral propensity to seek rewards despite the probability of negative consequences [43]. This construct represents an attitude that is independent of any probability of outcome, but is anchored in how individuals value those outcomes [44]. Research has shown the link between different levels of risk-taking propensities and decision making in many contexts. Though the importance of individual differences in decision making has been examined in other fields, little information security research has investigated the impact of individual traits, especially in phishing susceptibility as it relates to health care. Hansen et al [45] found that risk-taking propensity has a direct positive influence on behavioral intention in the context social media use.

The third study hypothesis (H3, Figure 1) was that risk-taking propensity is positively associated with phishing susceptibility.

#### Role of Sex

Differences between males and females in their online health information sharing behavior is another problem worth investigating. Male-female differences in behavior with regard to phishing has been mostly investigated in general but not specifically in health care–related phishing scenarios. For example, Sun et al [46] found that male students score better than female students in antiphishing behavior. Similarly, Verkijika [47] reported consistent findings in a general phishing context. Results are expected to be consistent in the health care phishing scenario as well.

The fourth study hypothesis (H4, Figure 1) was that males have lower phishing susceptibility than females.

### Data Collection

Data were collected online by administering the survey through Amazon Mechanical Turk (Amazon.com, Inc, Seattle, WA, USA), an online survey administration platform that allows for recruiting participants. The participants were redirected to take the survey built in Qualtrics^XM^ experience management software (Qualtrics). Participants had to be 18 years of age or older and reside in the United States. Many studies in the health care information technology context have used online data collection [27,48]. To measure phishing susceptibility (the dependent variable), participants were exposed to a scenario where they had to read an email and then indicate their intention to click on the email. The email was a phishing email in a health care context but participants were not told any information about its validity. The email was adopted from InfoSec [49].

### Data Description

Data were cleaned and recoded using SAS version 9.4 (SAS Institute Inc). Responses that included missing data were removed from the final dataset.

### Variables in the Model

All latent variables in the survey were borrowed from previous research and adapted to fit this study. Multimedia Appendix 1 shows the items and the source for variables in the model. All latent variable were measured using a Likert scale scored from 1 to 5. Sex was recoded to a binary variable named *Male* where 1 referred to a male participant and 0 referred to a female participant. Control variables were age, income, education, employment, and prior knowledge of the phishing concept.

### Measurement Model

After the data were cleaned using SAS version 9.4, IBM SPSS Amos version 25 (IBM Corporation) was used to assess the latent validity and reliability of the latent variables and the overall fit of the measurement model. Confirmatory factor analysis was used to evaluate the overall measurement model.

The validity of the variables was tested using the average variance extracted (AVE). Finally, multicollinearity was assessed using variable inflation factor.

## Results

### Participant Characteristics

After the data cleaning process, a total of 200 valid response were included in the study. Table 1 shows the descriptive statistics of the participants. For example, 43% of the participants are female and 57% are male.

**Table 1:** Descriptive statistics of participants (N=200).

Variable, Category		Values, n (%)
**Sex**		
	Female	86 (43.0)
	Male	114 (57.0)
**Age (years)**		
	18-25	25 (12.5)
	26-35	113 (56.5)
	36-45	31 (15.5)
	46-55	19 (9.5)
	>55	12 (6.0)
**Education**		
	High school or less	23 (11.5)
	Technical or community college	26 (13.0)
	4-year college degree	105 (52.5)
	Master’s degree	42 (21.0)
	Doctoral degree	2 (1.0)
	Other	2 (1.0)
**Income (US $)**		
	<25,000	41 (20.5)
	25,000 to <50,000	74 (37.0)
	50,000 to <75,000	58 (29.0)
	≥75,000	27 (13.5)

### Structural Equation Modeling

**Figure 2:**
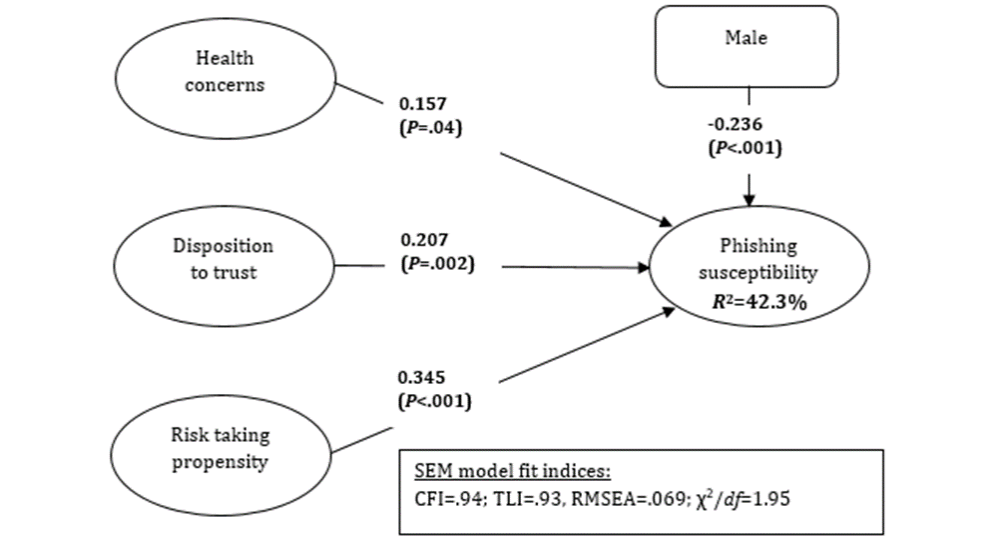
Structural equation modeling (SEM) results. CFI: comparative fit index; RMSEA: root mean square error of approximation; TLI: Tucker-Lewis index.

Structural equation modeling (SEM) was used to test the hypothesized model. Estimates derived from the SEM analysis were used to test the research hypotheses. The model explains 36.9% of the variance in phishing susceptibility. Figure 2 shows the SEM results for the hypothesized model.

Table 2 show the results of the confirmatory factor analysis. The results show that the model fit well: root mean square error of approximation was .061, comparative fit index was .98, and Tucker-Lewis index was .97 [50]. In addition, all item loadings were high and significant, with scores ranging between .75 and .95.

All scores exceeded .5 AVE, which meets the cutoff for establishing convergent validity [51]. In fact, the smallest AVE score was .62. All AVE scores exceeded the squared construct intercorrelation for the corresponding variable, thus establishing discriminant validity [51]. All variables were reliable, as the construct reliability scores ranged from .83 to .96, exceeding the cutoff score of .7 for establishing reliability of the variable [52] (Table 2).

All variable inflation factor scores were well below the cutoff value of 10 [53]. Thus, there was no evidence to suggest the existence of multicollinearity. In addition, all variables were conceptually distinct.

Hypothesis 1 proposed a positive relationship between health concerns and phishing susceptibility. The SEM results supported this hypothesis. The path coefficient for the health concerns was positive and significant (β=.157, *P*<.04). These results suggested that health concerns lead to higher phishing susceptibility.

Hypothesis 2 suggested that disposition to trust leads to higher phishing susceptibility. The results supported this hypothesis (β=.14, *P*=.04). The path coefficients for disposition to trust were positive and significant, which provides evidence to support hypothesis 2. The finding proposes that individuals who have a propensity to trust others are more susceptible to phishing attacks. In addition, the magnitude of the path coefficient is higher than that of health concerns, indicating a larger influence of disposition to trust than of health concerns on phishing susceptibility.

Hypothesis 3 posited a positive relationship between risk-taking propensity and phishing susceptibility. The results show that the path coefficient was positive and significant (β=.345, *P*<.001), suggesting that risk-taking propensity yields a higher phishing susceptibility, thus supporting hypothesis 3. In addition, the magnitude of the path coefficient is the highest of all antecedents.

Hypothesis 4 argued that males, compared with females, have a lower phishing susceptibility. This hypothesis was supported by the results. The path coefficient for negative and significant (β=–.236, *P*<.001). This result is consistent with previous research [47].

**Table 2:** Confirmatory factor analysis results.

Variable, item^a^		Loading	Construct reliability	Average variance extracted	Variable inflation factor
**Phishing susceptibility (PHS)**			**.959**	**.887**	**N/A^b^**
	PHS_1	.947			
	PHS_2	.947			
	PHS_3	.931			
**Health concerns (HC)**			**.832**	**.624**	**1.27**
	HC_1	.805			
	HC_2	.809			
	HC_3	.754			
**Disposition to trust (DTR)**			**.89**	**.73**	**1.23**
	DTR_1	.81			
	DTR_2	.9			
	DTR_3	.85			
**Risk-taking propensity (RT)**			**.904**	**.76**	**1.63**
	RT_1	.832			
	RT_2	.911			
	RT_3	.87			
**Measurement model goodness-of-fit indices**					
	RMSEA^c^	.061	N/A	N/A	N/A
	Comparative fit index	.98	N/A	N/A	N/A
	Tucker-Lewis index	.97	N/A	N/A	N/A
	χ^2^/*df*	1.737	N/A	N/A	N/A

^a^See Multimedia Appendix 1 for item details.

^b^N/A: not applicable.

^c^RMSEA: root mean square error of approximation.

## Discussion

### Principal Findings

This study investigated the relationship between personality-based factors and phishing susceptibility as it relates to health care. Personality-based factors have been shown to be associated with action, decision, attitude, and intention in various contexts [27,41,45]. However, limited research has focused on these relationship in a health care phishing context. This study focused on three main personality-based antecedents: health concerns, risk-taking propensity, and disposition to trust. This is one of the few studies that focused on these factors in the phishing context.

The study found that all three factors have in important role in leading to higher phishing susceptibility in a health care scenario. These findings suggest that personality-based factors should be taken into consideration when training individuals on phishing attacks and testing their phishing susceptibility and antiphishing behavior. The influence and effectiveness of training may differ based on personality-based traits. Thus, one-size-fits-all training, simulations, and strategy might not benefit most individuals.

While health concerns were found to be a motivator to engage patients in sharing their personal health information with providers [27], in a phishing scenario health concerns led to higher phishing susceptibility. Thus, health concerns could act as a “double-edged sword.” Of all personality-based factors, the results suggested that risk-taking propensity had the highest effect on increasing phishing susceptibility. This implies that future research should study the high risk taker to find optimal methods to reduce phishing susceptibility. The study also confirmed previous findings that females are more susceptible to phishing attacks, but this research confirmed the finding in a health care context.

### Limitations and Future Work

This study had several limitations, which can be addressed in future studies. The data were self-reported rather than actual behavior. Real-world behavior is very difficult to capture specifically in the health care context. In future studies, the influence of customized phishing training on individuals with different levels of personality-based factors will be tested.

### Conclusions

Phishing remains a problem that continues to increase. While companies, experts, and researchers continue to develop new methods to detect phishing attacks and improve resistance to falling a victim to phishing, attackers are advancing and improving phishing attacks at a higher and a more successful rate. Microsoft named 2019 as a year of phishing evolution as attackers made innovations in both technical and social tactics. The only way to mitigate phishing attacks is get ahead of the attackers. Training and simulation should include a balance of customized material and approaches that fit the characteristics of the receiver.

This study contributes to the phishing literature by investigating personality-based factors and reporting findings that are new and important. In addition, the study contributes to the health care information technology literature by examining health care–related phishing scenarios and factors. There is limited research in the health care context that deals with phishing susceptibility.

## Appendix

Multimedia Appendix 1 Measurement.

## Abbreviations

AVE: average variance extracted
SEM: structural equation modeling
