# Supplementary material for: The Role of Health Concerns in Phishing Susceptibility: Survey Design Study
Source: J Med Internet Res. 2020 May 4;22(5):e18394. doi: 10.2196/18394 (PMC7235804; doi:10.2196/18394)
Supplement: Multimedia Appendix 1 [file jmir_v22i5e18394_app1.docx]

**Appendix A**

**Table A1.** Measurement Items

| **Variable** | **Items** | **Questions** | **Adapted from** |
| --- | --- | --- | --- |
|  |  |  |  |
| Phishing Susceptibility | PHS_1 | I am likely to click on the link. | [1] |
|  | PHS_2 | I will probably click on the link. |  |
|  | PHS_3 | I am willing to click on the link. |  |
| Health Concerns | HC_1 | I always worry about my health. | [2] |
|  | HC_2 | I worry about my health more than other people worry about their health. |  |
|  | HC_2 | My health is a concern in my life. |  |
| Disposition to Trust | DTR_1 | I am a trusting person | [3] |
|  | DTR_2 | I trust people in general |  |
|  | DTR_3 | I think most people can be trusted |  |
| Risk Taking Propensity | RT_1 | I like to take risks. | [4] |
|  | RT_2 | Compared to most people I know, I like to “live life on the edge.” |  |
|  | RT_3 | Compared to most people I know, I like to gamble on things. |  |

| **Figure A1.** Phishing Email Shown to Participants |
| --- |
| **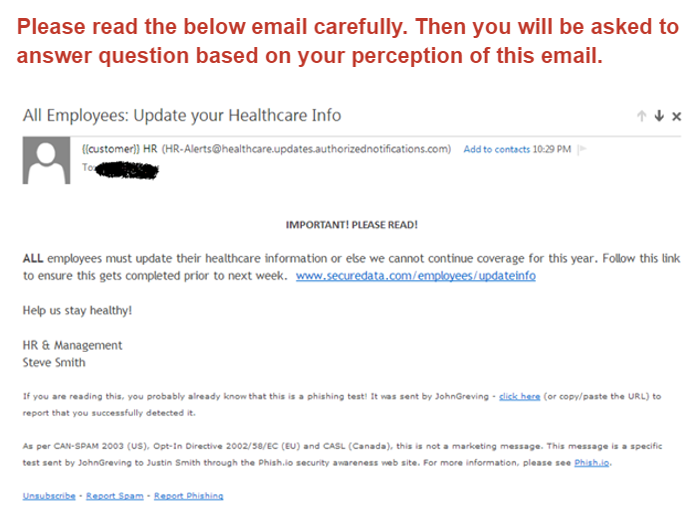** |

Based on the email they read, participants were asked to indicate what they would do:

| **Figure A2.** Example of question asked after viewing the email |
| --- |
| **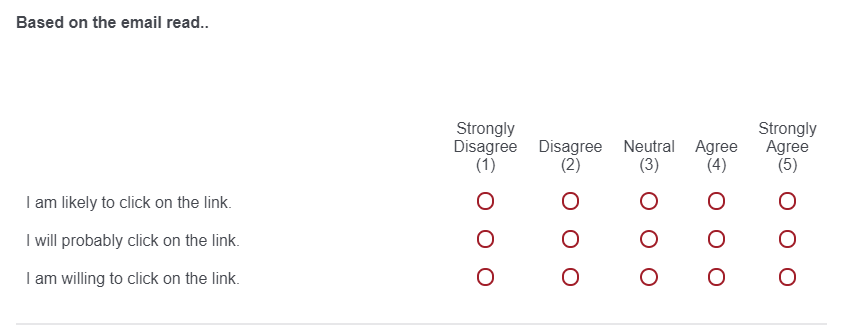** |

1. Malhotra, N.K., S.S. Kim, and J. Agarwal, *Internet users' information privacy concerns (IUIPC): The construct, the scale, and a causal model.* Information systems research, 2004. **15**(4): p. 336-355.

2. Jayanti, R.K. and A.C. Burns, *The antecedents of preventive health care behavior: An empirical study.* Journal of the academy of marketing science, 1998. **26**(1): p. 6-15.

3. Wu, G., X. Hu, and Y. Wu, *Effects of perceived interactivity, perceived web assurance and disposition to trust on initial online trust.* Journal of Computer-Mediated Communication, 2010. **16**(1): p. 1-26.

4. Burton, S., et al., *A scale for measuring attitude toward private label products and an examination of its psychological and behavioral correlates.* Journal of the academy of marketing science, 1998. **26**(4): p. 293.
